# Boredom-Driven Curious Learning by Homeo-Heterostatic Value Gradients

**DOI:** 10.3389/fnbot.2018.00088

**Published:** 2019-01-22

**Authors:** Yen Yu, Acer Y. C. Chang, Ryota Kanai

**Affiliations:** Araya, Inc., Tokyo, Japan

**Keywords:** curiosity, boredom, goal-directedness, intrinsic motivation, outcome devaluation, satiety, homeostatic motivation, heterostatic motivation

## Abstract

This paper presents the Homeo-Heterostatic Value Gradients (HHVG) algorithm as a formal account on the constructive interplay between boredom and curiosity which gives rise to effective exploration and superior forward model learning. We offer an instrumental view of action selection, in which an action serves to disclose outcomes that have intrinsic meaningfulness to an agent itself. This motivated two central algorithmic ingredients: devaluation and devaluation progress, both underpin agent's cognition concerning intrinsically generated rewards. The two serve as an instantiation of homeostatic and heterostatic intrinsic motivation. A key insight from our algorithm is that the two seemingly opposite motivations can be reconciled—without which exploration and information-gathering cannot be effectively carried out. We supported this claim with empirical evidence, showing that boredom-enabled agents consistently outperformed other curious or explorative agent variants in model building benchmarks based on self-assisted experience accumulation.

## 1. Introduction

In this study, we present an instrumental view of action selection, in which an action serves to disclose outcomes that have intrinsic meaningfulness—i.e., that hold epistemic values—to an agent itself. The implication of this statement is twofold: (1) for agents whose innate goal appeals to their own knowledge gain, the occurrence of curiosity rests upon the devaluation of known knowledge (and hence goal-directedness); (2) boredom—consequential to devaluation—and curiosity entail a mutually reinforcing cycle for such kind of (meaningful) disclosure to ensue.

Animal studies have shown that learning stimulus-response (S-R) associations through action-outcome reinforcement is but one facet of instrumental behavior. Internally, animals may build models that assign values to reappraise experienced outcomes. This expands the landscape of instrumental behavior to include the stimulus-outcome-response (S-O-R) learning system—or goal-directed learning (Balleine and Dickinson, 1998). Goal-directed behavior is known in both empirical and computational approaches to support adaptive and optimal action selection (Adams and Dickinson, 1981; Adams, 1982; Mannella et al., 2016). Central to such behavioral adaptiveness is devaluation. This means for a given action-outcome pair the associated reinforcing signal is no longer monotonic. Instead, an outcome value will change with reappraisals in accordance with an agent's internal goal.

One classic paradigm of devaluation manipulates an agent's level of satiation based on food accessibility, leading to altered behavioral patterns. In the context of epistemic disclosure, an analogy can be drawn between devaluation and the emergence of boredom, in which one's assimilation of knowledge reduces the value of similar knowledge in future encounters. The relationship between boredom and outcome devaluation has a long history in psychological research. Empirical findings indicated that boredom is reportedly accompanied by negative affective experiences, suggesting that experienced outcomes are intrinsically evaluated and considered as less valuable (Perkins and Hill, 1985; Vodanovich et al., 1991; Fahlman et al., 2009; van Tilburg and Igou, 2012; Bench and Lench, 2013).

Psychophysiological studies also demonstrated that boredom plays an active role in eliciting information-seeking behaviors. Subjects showing higher levels of reported boredom are accompanied by increased autonomic arousal, such as heart rate and galvanic skin response. These findings are in line with our key notion that boredom intrinsically and actively drives learning behaviors (Berlyne, 1960; London et al., 1972; Harris, 2000). Note, however, that this notion is contested and a matter of unsettled debate (e.g., Eastwood et al., 2012; Fahlman et al., 2013; Merrifield and Danckert, 2014; Danckert et al., 2018). It is therefore worth pointing out that boredom may be accompanied by a low arousal state (Barmack, 1939; Geiwitz, 1966; Mikulas and Vodanovich, 1993; Pattyn et al., 2008; Vogel-Walcutt et al., 2012).

A finding by Larson (1990) invites the speculation that a task set may interact with boredom, thereby modifying a subject's behavioral pattern to follow either low or high arousal states. This means boredom may merely signal a state of disengagement. Whether an agent's cognitive resources can be freely allocated to re-engage another task inherently depends upon the existence of a prohibiting condition. Larson's (1990) participants, who reported boredom and were later rated with low scores in creative writing, were by design not allowed to disengage from the essay-writing task. Other theories, on the other hand, suggested that boredom is associated with increase in creativity (Schubert, 1977, 1978; Harris, 2000).

We thus postulate that, in the absence of any *a priori* cognitive or behavioral constraints, a state of boredom is followed by an attempt to diversify one's experience. That is, boredom begets exploration. This is in line with Vodanovich and Kass's (1990) notion of boredom in “inspiring a search for change and variety” and Zuckerman's (2008) “sensation-seeking.” Sensation-seeking (Zuckerman, 1971, 2008; Kass and Vodanovich, 1990; Dahlen et al., 2005) is categorized as a personality trait, tightly linked to boredom susceptibility (Zuckerman et al., 1978). High sensation seekers get bored more easily, suggesting that individuals susceptible to boredom are predisposed to seek novel sensations. As a result, a learner who is also a novelty-seeker may have a world model that generalizes better. In our framework, receiving novel sensations is formalized as planning to visit states where an agent can effectively learn faster (i.e., the agent gets bored quicker). This effect is then treated as an intrinsic reward, prompting an agent to continue experiencing the state before the reward is depleted.

A recent computational modeling tapped into a similar theme (Gomez-Ramirez and Costa, 2017), where boredom facilitates exploration. However, our work differs from that of Gomez-Ramirez and Costa (2017) in that our model permits a simple form of agency (by having an action policy) and focuses on learning. Additionally, their exploration may favor predictable state space, whereas our agent will treat high predictability as an intrinsically non-rewarding state.

Finally, in psychology studies, the term boredom usually comes under two distinct constructs: a state of boredom and boredom proneness (Elpidorou, 2014, 2017; Mugon et al., 2018). Boredom proneness is regarded as the psychological predisposition of an individual to experience boredom which poses a systematic impact on one's social and psychological well-being. By contrast, a state of boredom is seen as a transient, regulatory signal that prompts one's behaviors into alignment with its goal-directedness (Elpidorou, 2017). In this sense, our model conceptually encompasses the function of the state boredom regulatory signal.

Curiosity, irrespective of being a by-product of external goal-attainment or an implicit goal in and of an agent itself, is often ascribed as a correlate of information-seeking behavior (Gottlieb et al., 2013). Behaviors exhibiting curious quality are observed in humans and animals alike, suggesting an universal role of curiosity in shaping one's fitness in terms of survival chance. Though the exact neural mechanism underlying the emergence of curious behavior still remains obscure, current paradigms have their focus on (1) novelty disclosure and (2) uncertainty reduction aspects of information-seeking (Bellemare et al., 2016; Friston et al., 2017; Ostrovski et al., 2017; Pathak et al., 2017). Indeed, both aspects can be argued to improve agent's fitness in epistemic landscape if the agent elects to incorporate the novelty or uncertainty.

Both boredom and curiosity are tightly connected to the notion of intrinsic motivation. Specifically, the occurrence of boredom and curiosity can be mapped to homeostatic and heterostatic motivations, respectively. The homeostatic and heterostatic motivations as two important classes of intrinsic motivation have been extensively reviewed in Oudeyer and Kaplan (2009). Simply, a homeostatic motivation drives a system to compensate perturbations in order to reach some equilibrial state. A heterostatic motivation is the opposite of a homeostatic motivation. A system that is driven by heterostatic motivations will self-perturb out of its equilibrium. In our formalism, predictive model learning and policy learning, each respectively induces boredom and curiosity, suggesting that the two classes of motivation can in fact be complementary when the two learning tasks are carried out concurrently. Our contribution thus pertains to the reconciliation of homeo-heterostatic motivations.

## 2. Markov Decision Process

In what follows, we briefly review preliminaries for the ensuing algorithm. We focus on well-established themes surrounding typical reinforcement learning, including Markov Decision Process and value gradients as a policy optimisation technique.

In Markov Decision Process (MDP) one considers the tuple (*S, A, R, P*, π, γ). *S* and *A* are spaces of real vectors whose member, ***s*** ∈ *S* and ***a*** ∈ *A*, represent states (or sensor values) and actions. *R* is some reward function defining the mapping *R*:*S* × *A* → ℝ. The probabilities associated with states and actions are given by the forward model *P*(*S*′|*A* = ***a***, *S* = ***s***) and the action policy π(*A*|*S* = ***s***). Throughout the paper we use the ‘primeș notation, e.g., ***s***′, to represent one time step into the future: ***s***′ = ***s***(*t* + 1).

The goal of MDP is to optimally determine the action policy π^*^ such that the expected cumulative reward over a finite (or infinite) horizon is maximized. Considering a finite horizon problem with discrete time, *t* ∈ [0, *T*], this is equivalent to $\pi^{*}=\;\arg\max_{\pi }E_{a\sim \pi }\left[\sum_{t=0}^{T}\gamma^{t}R(s(t),\;a(t))\right]$, where γ ∈ [0, 1] is the discount factor.

Many practical approaches for solving MDP often resort to approximating state-action value *q*(***a***, ***s***) or state value *v*(***s***) functions (Sutton and Barto, 1998; Mnih et al., 2013; Heess et al., 2015; Lillicrap et al., 2015). These value functions are given in the Bellman equation

(1) $$\begin{array}{l} v(s)=E_{\pi (a|s)}\left[R(a,s)+\gamma q(a,s)\right]\\ \;=E_{\pi (a|s)}\left[R(a,s)+\gamma E_{P(s^{\prime }|a,s)}[v(s^{\prime })]\right] \end{array}$$

When differentiable forward model and reward function are both available, policy gradients can be analytically estimated using value gradients (Fairbank and Alonso, 2012; Heess et al., 2015).

## 3. Homeo-Heterostatic Value Gradients

This section describes formally the algorithmic structure and components of the Homeo-Heterostatic Value Gradients, or HHVG. The naming of HHVG suggests its connections with homeostatic and heterostatic intrinsic motivations. A detailed review on homeostatic and heterostatic motivations are given in Oudeyer and Kaplan (2009). Briefly, a homeostatic motivation encourages an organism to occupy a set of predictable, unsurprising states (i.e., a *comfort zone*). Whereas, a heterostatic motivation does the opposite; curiosity belongs to this category.

The algorithm offers a reconciliation between the two seemingly opposite qualities and concludes with their cooperative nature. Specifically, the knowledge an organism maintains about its comfort zone helps instigate outbound heterostatic drives. In return, satisfying heterostatic drives broadens the organism's extent of comfort zone. As a consequence, the organism not only improves its fitness in terms of homeostatic outreach but also becomes effectively curious.

### 3.1. Nomenclature and Notations

It is instructive to overview the nomenclature of the algorithm. We consistently associate homeostatic motivation with the emergence of *boredom*, which reflects the result of having incorporated novel information into one's knowledge, thereby diminishing the novelty to begin with. This is conceptually compatible with outcome *devaluation* or induced satiety in instrumental learning. *Devaluation progress* is therefore referred to as one's epistemic achievement. That is, the transitioning of a priori knowledge to one of having assimilated otherwise unknown information. The devaluation progress is interpreted as an instantiation of intrinsic reward. The drive to maintain steady rewards conforms to a heterostatic motivation.

The notation L(·) consistently denotes loss functions throughout the paper; any variables on which the loss function depends are always made explicit. There are occasions where we abbreviated the loss function to avoid clutters. A definition such as $L_{mm}\left(\psi \right):=L\left(\text{a},\text{s};\psi ,\theta \right)$ is then given upon first appearance. Here, the subscript *mm* indicates *meta-model*. One may tell in this example that the symbols ***a***, ***s***, and θ on the right hand side are temporarily omitted. This means the optimisation procedure for the meta-model concerns only the parameter ψ. Similarly, this applies to $L_{fm}$, $L_{vf}$, and $L_{ap}$, where the subscripts stand for *forward model, value function*, and *action policy*. The symbol N is reserved for Normal distribution.

### 3.2. Intuition

**Figure 1:**
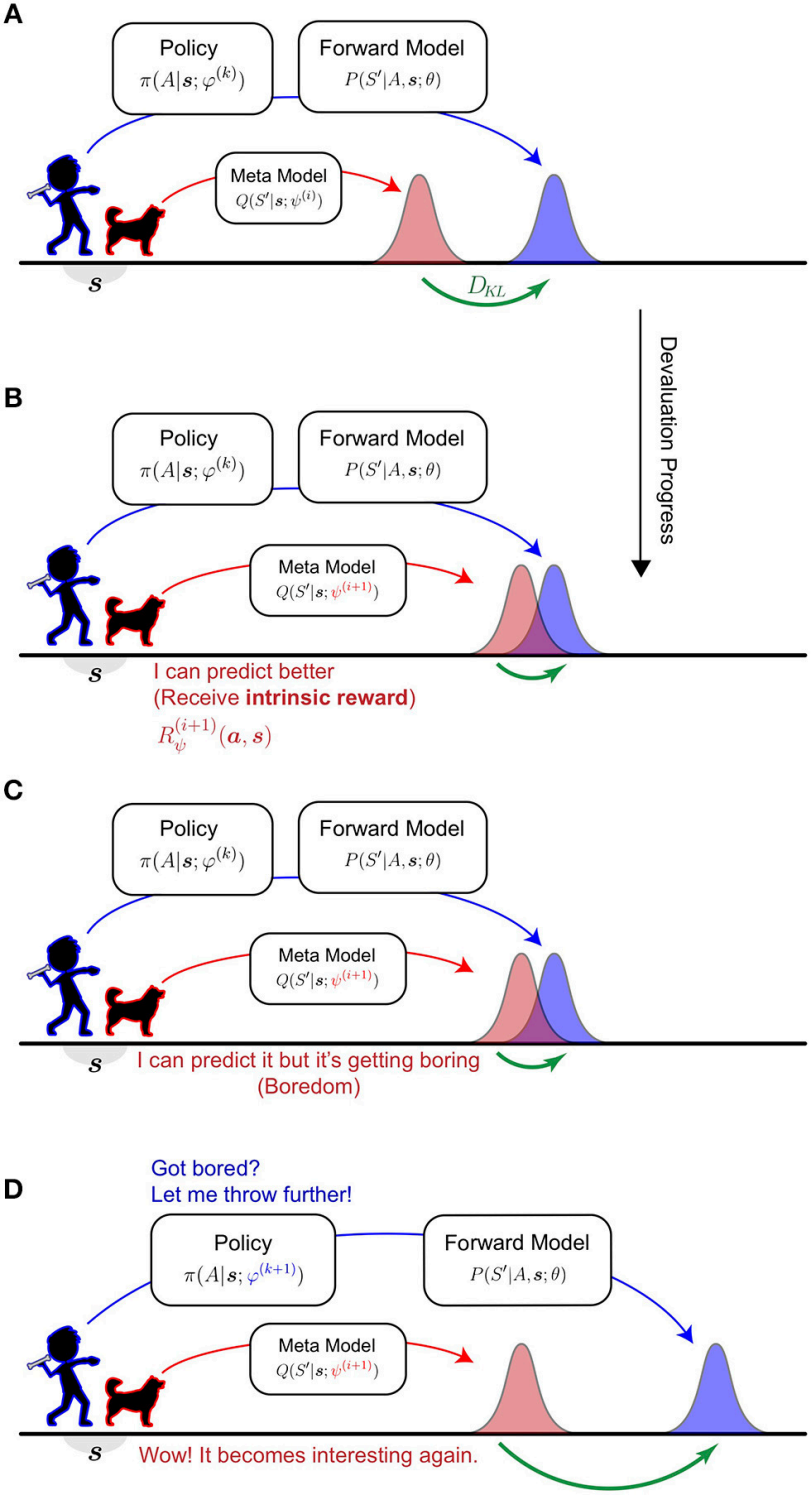
Intuitive understanding of the Homeo-Heterostatic Value Gradients (HHVG) algorithm. **(A)** The algorithm can be interpreted as the cooperative interplay between a thrower (kid; blue) and a catcher (dog; red). The thrower is equipped with a forward model that estimates its aiming and is controlled by an action policy. Without knowing the thrower's policy, the catcher (meta-model), in order to make good catches, infers where the thrower is aiming on average. **(B)** The catcher is interested in novel, unpredicted throws. Whenever the catcher improves its predictive power some intrinsic reward (devaluation progress) is generated. **(C)** As the catcher progresses further, similar throws become highly predictable, thus inducing a sense of boredom. **(D)** To make the interplay interesting again, the thrower is driven to devise new throws, so that the catcher can afford to make further progress. By repeating **(A,B)** the thrower has attempted diverse throws and known well about its aim. At the same time, the catcher will assume a vantage point for any throw.

An intuitive understanding of HHVG is visualized in Figure 1. Imagine the interplay between a thrower and their counterpart—a catcher. The catcher anticipates where the thrower is aiming and makes progress by improving its prediction. The thrower, on the other hand, keeps the catcher engaged by devising novel aims. Over time, the catcher knows well what the thrower is capable of, whilst the thrower has attempted a wide spectrum of pitches.

In the algorithm, the thrower is represented by a forward model attached to a controller (policy) and the catcher a “meta-model.” We unpack and report them individually. Procedural information is summarized in Algorithm 1.

**Algorithm 1:**
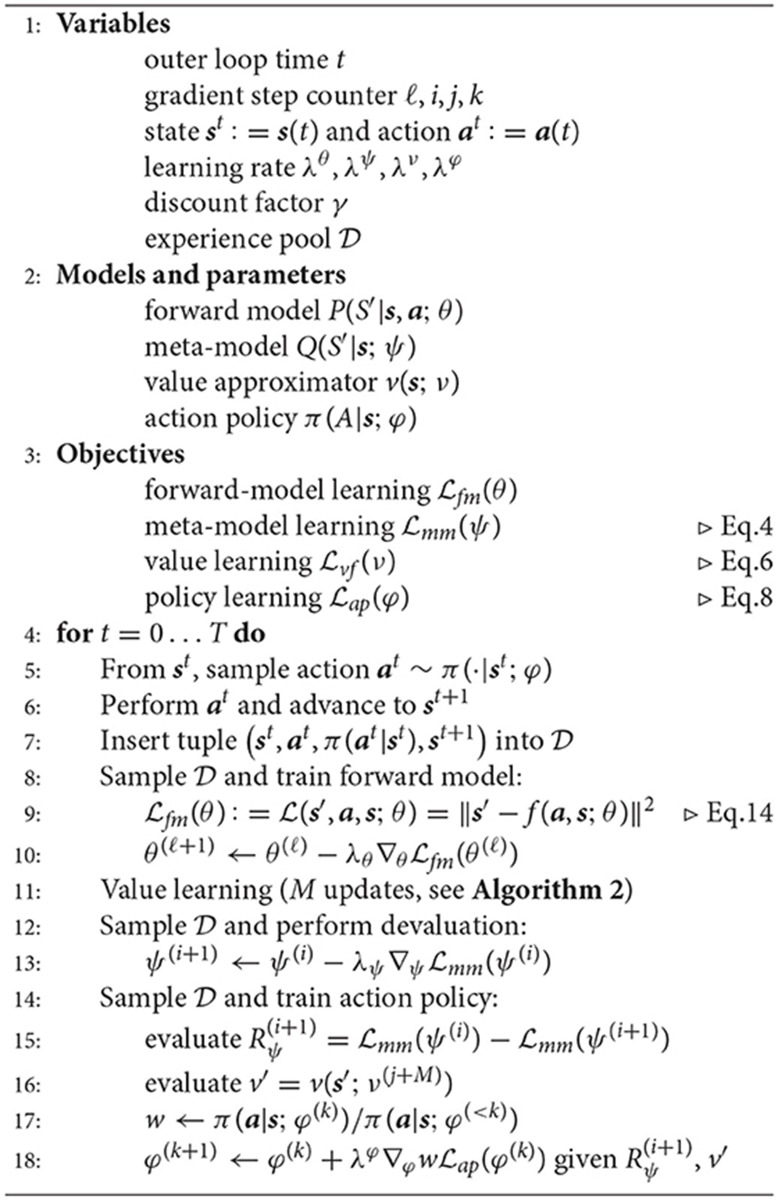
Homeo-heterostatic value gradients

**Algorithm 2:**
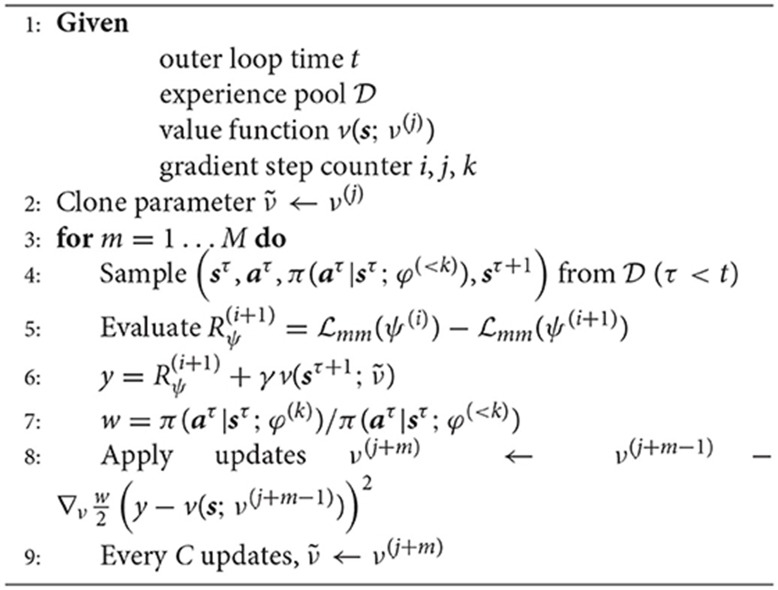
Fitted Policy Evaluation [cf. Heess et al. (2015)]

### 3.3. Forward Model

We start by specifying at current time the state and action sample as ***s*** and ***a***. The forward model describes the probability distribution over future state *S*′, given ***s***, ***a***, and parameter θ.

(2) $$P(S^{\prime }|A=a,S=s;\theta )$$

The entropy associated with *S*′, conditioned on ***s*** and ***a***, gives a measure of the degree to which *S*′ is informative on average. We referred to this measure as one of *interestingness*. Note this is a different concept from the “interestingness” proposed by Schmidhuber (2008), which is the first-order derivative of compressibility.

### 3.4. Boredom, Outcome Devaluation, and Meta-Model

Boredom, in common understanding, is perhaps not unfamiliar to most people under the situation of being exposed to certain information which one has known well by heart. It is the opposite of being interested. In the current work, we limited the exposure of information to those being disclosed by one's actions.

To mark the necessity of boredom, we first identify the limitation of a naive instantiation of curiosity; then, we show that the introduction of boredom serves to resolve this limitation.

Consider the joint occurrence of future state *S*′ and action *A*: *P*(*S*′, *A*|*S* = ***s***; θ, φ). This can be derived from the product rule of probability using *P*(*S*′|*A* = ***a***, *S* = ***s***; θ) (as shown Equation 2) and action policy π(*A*|*S* = ***s***; φ), parametrised by φ (action policy is revisited in section 3.6).

A naive approach to curiosity is by optimizing the action policy, such that *A* is predictive of maximum *interestingness* (see section 3.3) about the future.

However, this naive approach would certainly lead to the agent behaving habitually and, as a consequence, becoming obsessive about a limited set of outcomes. In other words, a purely interestingness-seeking agent is a darkroom agent (see section 3.7; also Friston et al., 2012 for related concept).

Such obsession with limited outcomes poses a caveat—the agent has no recourse to inform itself about prior exposure of similar sensations. If the agent is otherwise endowed with this capacity, namely, by assimilating previous experiences into summary statistics, an ensuing sense of boredom would be induced. The induction of boredom essentially causes the agent to value the same piece of information less, thus changing the agent's perception toward interestingness. If the agent were to pursue the same interestingness-seeking policy, a downstream effect of boredom would drive the agent to seek out other information that could have been known. This conception amounts to an implicit goal of *devaluating* known outcomes.

To this end, we introduce the following meta-model *Q* to represent *a priori* knowledge about the future. Note that *Q* is a conditional probability function over *S*′ and is not to be confused with a state-action value function *q*(***a***, ***s***) in MDP. The meta-model, parametrised by ψ, is an approximation to the *true* marginalization of joint probability *P*(*S*′, *A*|*S* = ***s***; θ, φ) over *A*:

(3) $$\begin{array}{l} Q(S^{\prime }|S=s;\psi )\approx P(S^{\prime }|S=s;\theta ,\varphi )\\ \;=\sum_{A}\left[P(S^{\prime },A|s;\theta ,\varphi )\right]\\ \;=\sum_{A}\left[P(S^{\prime }|A,s;\theta )\pi (A|s;\varphi )\right] \end{array}$$

We associate the occurrence of boredom, or, synonymously, outcome devaluation, with minimizing the devaluation objective with respect to ψ. The devaluation objective is given by the Kullback-Leibler (KL) divergence:

(4) $$\begin{array}{l} L_{mm}(\psi ):=L(a,s;\psi ,\theta )\\ \;=D_{KL}\left[P(s^{\prime }|a,s;\theta )\|Q(s^{\prime }|s;\psi )\right] \end{array}$$

### 3.5. Devaluation Progress, Intrinsic Reward, and Value Learning

Through the use of KL-divergence in Equation 3, we emphasize the complementary nature of devaluation in relation to a knowledge-gaining process. That is to say, devaluation results in information gain for the agent. This, in fact, can be regarded as cognitively rewarding and, thus, serves to motivate our definition of intrinsic reward.

One rewarding scenario happens when *Q*(*S*′|***s***; ψ) has all the information there is to be possessed by *A* about *S*′. *A* is therefore rendered redundant. One may speculate, at this point, the agent could opt for inhibiting its responses. Disengaging actions potentially saves energy which is rewarding in biological sense. This outcome is in line with the “opportunity cost model” proposed by Kurzban et al. (2013). In their model, boredom is seen as a resource regulatory signal which drives an agent to disengage the current task and curb the computational cost. As a consequence, the occurrence of boredom may encourage re-allocation of computational processes to alternative higher-value activities (Kurzban et al., 2013).

Alternatively, the agent may attempt to develop new behavioral repertoires, bringing into *S*′ new information (i.e., novel outcomes) that is otherwise unknown to *Q*. The ensuing sections will focus on this line of thinking.

From Equation 4, we construct the quantity *devaluation progress* to represent an intrinsically motivated reward. The devaluation progress is given by the difference between KL-divergences before and after devaluation [as indicated by the superscript (*i* + 1)]:

(5) $$\begin{array}{l} R_{\psi }^{\left(i+1\right)}(a,s):=L(a,s;\psi^{\left(i\right)},\theta )-L(a,s;\psi^{\left(i+1\right)},\theta )\\ \;=L_{mm}(\psi^{\left(i\right)})-L_{mm}(\psi^{\left(i+1\right)}), \end{array}$$

Here, we write $R_{\psi }^{\left(i+1\right)}\left(\text{a},\text{s}\right)$ in accordance with notational convention in reinforcement learning, where reward is typically a function of state and action. Subscript ψ indicates the dependence of *R* on meta model parameter.

Having established the intrinsic reward, value learning is such that the value function approximator *v*(***s***; ν) follows the Bellman equation $v\left(\text{s}\right)=E_{\text{a}}\left[R\left(\text{a},\text{s}\right)+\gamma E_{{\text{s}}^{\prime }}\left[v\left({\text{s}}^{\prime }\right)\right]\right]$. In practice, we minimize the objective with respect to ν:

(6) $$\begin{array}{l} L_{vf}(\nu ):=L(s^{\prime },a,s;\nu )\\ \;=\|y-v(s;\nu ){\|}^{2}\\ \;y=R_{\psi }^{\left(i+1\right)}(a,s)+\gamma v(s^{\prime };\tilde{\nu }) \end{array}$$

### 3.6. Policy Optimisation

We define action policy at state *S* = ***s*** as the probability distribution over *A* with parameter φ:

(7) π(A|S=s;φ)

Our goal is to determine the policy parameter φ that maximizes the expected sum of future discounted rewards. One approach is by applying Stochastic Value Gradients (Heess et al., 2015) and maximizes the value function. We thus define our policy objective as follows (notice the negative sign; we used a gradient update rule that defaults to minimization):

(8) $$\begin{array}{l} L_{ap}(\varphi ):=L(s^{\prime },a,s;\theta ,\psi^{\left(i\right)},\psi^{\left(i+1\right)},\nu ,\varphi )\\ \;=-E_{a\sim \pi (\cdot |s;\varphi )}\left[R_{\psi }^{\left(i+1\right)}(a,s)+\gamma E_{s^{\prime }\sim P(\cdot |a,s;\theta )}[v(s^{\prime };\nu )]\right] \end{array}$$

### 3.7. Remarks on Homeostatic and Heterostatic Regulations

Oudeyer and Kaplan (2009) outlined the distinctions between two important classes of intrinsic motivation: homeostatic and heterostatic. A homeostatic motivation is one that can be satiated, leading to a certain equilibrium behaviorally; whereas a heterostatic motivation topples the agent, thus preventing it from occupying habitual states.

Our algorithm entails regulations relating to both classes of intrinsic motivation. Specifically, the devaluation objective (Equation 3) realizes the homeostatic aspect due to its connection with induced satiety. On the other hand, the devaluation progress (Equation 4) introduced for policy optimisation instantiates a heterostatic drive to agent's behavioral pattern.

Heterostasis is motivated by the agent pushing itself toward novelty and away from devalued, homeostatic states (as revealed at the end of this section in Equation 12). This statement is shown formally by replacing the reward $R_{\psi }^{\left(i+1\right)}\left(\text{a},\text{s}\right)$ in Equation 7, with Equation 4. We then arrived at the following form involving expected KL-divergence:

(9) $$\begin{array}{l} -\;E_{a\sim \pi (\cdot |s;\varphi )}[D_{KL}[P(s^{\prime }|a,s;\theta )\|Q(s^{\prime }|s;\psi^{\left(i\right)})]\\ -\;D_{KL}[P(s^{\prime }|a,s;\theta )\|Q(s^{\prime }|s;\psi^{\left(i+1\right)})]]\\ -\;E_{a\sim \pi (\cdot |s;\varphi )}E_{s^{\prime }\sim P(\cdot |a,s;\theta )}\left[v(s^{\prime };\nu )\right]\\ =-\{I(S^{\prime }:A|S=s;\psi^{\left(i\right)},\varphi ,\theta )-I(S^{\prime }:A|S=s;\psi^{\left(i+1\right)},\varphi ,\theta )\\ +\;E_{a\sim \pi (\cdot |s;\varphi )}E_{s^{\prime }\sim P(\cdot |a,s;\theta )}\left[v(s^{\prime };\nu )\right]\} \end{array}$$

Notice that the expected devaluation progress becomes the difference between conditional mutual information *I* before (ψ^(*i*)^) and after devaluation (ψ^(*i*+1)^).

Assume, for the moment, that the agent is equipped with devaluation capacity only. In other words, we replace the devaluation progress and fall back on devaluation objective, $R:=L_{mm}\left(\psi \right)$ (cf. Equation 4). The agent is now interestingness-seeking with homeostatic regulation. We further suppose that the dynamics of ψ and φ evolve in tandem, which gives

(10) $$\begin{array}{l} I(S^{\prime }:A|S=s;\psi^{\left(i\right)},\varphi^{\left(k\right)})\to I(S^{\prime }:A|S=s;\psi^{\left(i+1\right)},\varphi^{\left(k\right)})\\ \;\to I(S^{\prime }:A|S=s;\psi^{\left(i+1\right)},\varphi^{\left(k+1\right)})\\ \;\to I(S^{\prime }:A|S=s;\psi^{\left(i+2\right)},\varphi^{\left(k+1\right)})\to \ldots \end{array}$$

In practice, the nature of devaluation and policy optimisation often depends on replaying agent's experience. Taking turn applying gradient updates to ψ and φ creates a self-reinforcing cycle that drives the policy to converge toward a point mass. For instance, if the policy is modeled by some Gaussian distribution, this updating scheme would result in infinite precision (zero spread).

For curiosity, however, such parameter dynamics should not be catastrophic if we subsume the homeostatic regulation and ensure the preservation of the relation given in Equation 11:

(11) $$\begin{array}{l} \;I(S^{\prime }:A|S=s;\psi^{\left(i+1\right)},\varphi^{\left(k\right)})\leq I(S^{\prime }:A|S=s;\psi^{\left(i\right)},\varphi^{\left(k\right)})\\ \;\leq I(S^{\prime }:A|S=s;\psi^{\left(i+1\right)},\varphi^{\left(k+1\right)})\\ \Rightarrow -I(S^{\prime }:A|S=s;\psi^{\left(i+1\right)},\varphi^{\left(k\right)})+I(S^{\prime }:A|S=s;\psi^{\left(i\right)},\varphi^{\left(k\right)})\\ \;\leq I(S^{\prime }:A|S=s;\psi^{\left(i+1\right)},\varphi^{\left(k+1\right)}) \end{array}$$

This equation holds because the devaluation process on average has a tendency to make *A* less informative about *S*′, after which *A* is perturbed to encourage a new *S*′ less predictable to *Q*. By rearranging the equation such that the left hand side remains positive, we have arrived at a lower bound on *I*(*S*′:*A*|*S* = ***s***; ψ^(*i*+1)^, φ^(*k*+1)^) which recovers the expected devaluation progress.

Equation 12 summarizes the argument associated with Equations (10, 11).

(12) $$\begin{array}{l} \varphi^{\left(k+1\right)}=arg\;\underset{\varphi^{\left(k\right)}}{max}[I(S^{\prime }:A|S=s;\psi^{\left(i\right)},\varphi^{\left(k\right)})\\ \;-\underset{{\bar{\psi }}^{\left(i\right)}}{\min}I(S^{\prime }:A|S=s;{\bar{\psi }}^{\left(i\right)},\varphi^{\left(k\right)})]\\ \;\neq arg\;\underset{\varphi^{\left(k\right)}}{max}\left[\underset{{\bar{\psi }}^{\left(i\right)}}{\min}I(S^{\prime }:A|S=s;\psi^{\left(i\right)},\varphi^{\left(k\right)})\right] \end{array}$$

Finally, we offer an intuition on how policy optimisation gives rise to heterostatic motivation. This is made clear from the optimized target *I*(*S*′:*A*|*S* = ***s***; ψ^(*i*+1)^, φ^(*k*+1)^), found on the right hand side of Equation 10. It is instructive to re-introduce the true marginalization *P*(*S*′|*S* = ***s***; θ, φ) from Equation 3; write:

(13) $$\begin{array}{l} I(S^{\prime }:A|S=s;\psi^{\left(i+1\right)},\varphi^{\left(k+1\right)})=\sum_{a}\pi (a|s;\varphi^{\left(k+1\right)})\\ \sum_{s^{\prime }}P(s^{\prime }|s,a;\theta )\log\frac{P(s^{\prime }|a,s;\theta )}{Q(s^{\prime }|s;\psi^{\left(i+1\right)})}=\sum_{a}\pi (a|s;\varphi^{\left(k+1\right)})\\ \sum_{s^{\prime }}P(s^{\prime }|s,a;\theta )\log\frac{P(s^{\prime }|a,s;\theta )}{P(s^{\prime }|s;\theta ,\varphi^{\left(k+1\right)})}\frac{P(s^{\prime }|s;\theta ,\varphi^{\left(k+1\right)})}{Q(s^{\prime }|s;\psi^{\left(i+1\right)})}\\ =I(S^{\prime }:A|S=s;\varphi^{\left(k+1\right)})+D_{KL}\left[P(s^{\prime }|s;\theta ,\varphi^{\left(k+1\right)})\|Q(s^{\prime }|s;\psi^{\left(i+1\right)})\right] \end{array}$$

Simply, the optimized policy is such that the agent increases the conditional mutual information and is pushed away (via increasing the KL-divergence) from its homeostatic state *Q*.

## 4. Implementation Considerations

This section presents practical considerations when motivating the aforementioned agent using neural networks. These considerations were mainly for the ease of calculating KL-divergence analytically.

### 4.1. Forward Model

We assumed that the state follows some Gaussian distribution with mean ***s*** and covariance Σ. The future state is described by its mean ***s***′ according to the deterministic mapping ***s***′ = *f*(***a***, ***s***; θ), where ***a*** is the action sampled from policy. *f* represents a neural network with trainable parameter θ:

(14) $$f(a,\;s;\theta )=As+\left(\sum_{\iota }a_{\iota }\;B^{\iota }\right)\;s+Ca+o$$

***A***, **B**, and **C** are approximations of Jacobian matrices and *o* a constant, all depending on θ. **B** is a three-way tensor indexed by ι along the first axis. This treatment is similar to Watter et al. (2015) (also cf. Karl et al., 2016), except that we considered a bilinear approximation and that, in the following sections, we used only the mean states in a deterministic environment.

The above formalism follows that ***s***′ has covariance matrix 𝔼[***s***′***s***′^**⊺**^] = ***J*****Σ*****J***^**⊺**^, where $\text{J}=\left(\text{A}+\underset{\iota }{\sum }a_{\iota }{\text{B}}^{\iota }\right)$. The transition probability is then given by

(15) $$P(S^{\prime }|A=a,S=s;\theta )=N(f(a,s;\theta ),\;J\Sigma J^{⊺})$$

The model parameter θ represented four fully connected layers of width 512; the four layers were complemented by a residual connection, which was a single fully connected layer. We used rectified linear units (ReLU) as output nonlinearities. Next, four fully connected, linear layers each mapped the 512-dimensional output into vectors of dimension 16, 32, 8, and 1. These vectors were then reshaped into tensors and used as ***A***, ***B***, ***C***, and *o*.

### 4.2. Meta Model

Our meta model was defined as a Gaussian distribution $Q\left(S^{\prime }|S=\text{s};\psi \right)=N\left(\mu^{\prime },\Sigma^{\prime };\psi \right)$, where the mean **μ**′ and covariance matrix **Σ**′ are outputs of a neural network parametrized by ψ. Specifically, to construct the covariance matrix, we used the fact that the eigendecomposition of a positive semi-definite matrix always exists. This then means we can use neural networks to specify an orthogonal matrix ***H*** and a diagonal matrix ***D***, such that the covariance matrix is equivalent to:

(16) $$\begin{array}{l} \Sigma^{\prime }=HDH^{⊺},\;D=\text{diag}(d)\\ \;H=I-2\frac{uu^{⊺}}{\|u{\|}^{2}}, \end{array}$$

where ***d*** is a positive-valued vector that specifies the diagonal elements of ***D***. The second line of Equation 15 shows how an orthogonal matrix can be built from a real-valued vector ***u***, called Householder vector (Tomczak and Welling, 2016). ***I*** is an identity matrix.

The network architecture used to compute μ′, ***d***, and ***u*** consisted of three trainable layers, each of which was identically structured. Three fully connected layers with ReLU activation functions, complemented by a residual connection, were followed by a linear, fully connected output layer. The output layer for ***d*** used a Softplus nonlinearity to ensure positive values.

We can, of course, let the neural network output a full matrix ***X*** and have **Σ**′ = ***XX***^**⊺**^. However, our method is less costly when scaling up the problem dimension.

### 4.3. Policy and Value Functions

Both the policy and value functions were identically structured in terms of network architecture. They consisted of four fully connected layers with ReLU activation functions, complemented by a residual connection. This was then followed by a linear output layer. The outputs for the policy network were treated as logits of a categorical distribution over action space.

## 5. Experiment

One testable hypothesis that emerges from our previous remark—that boredom gives rise to novelty seeking policy (cf. KL-divergence term in Equation 12)—is that *boredom helps improve agent's forward model learning*. This is because novelty seeking essentially implies diversity in agent's experience. In other words, a boredom-driven curious agent must exhibit a tendency toward *exploration* and against *perseveration*. This tendency is critical when the agent was not given a training set (on which it based its forward model learning) but has to self-assist in accumulating one from scratch.

Briefly, an agent that tends to explore would appear to accumulate experience that reflects a more complete picture of the environment and, therefore, leads to a more accurate forward model. By contrast, if an agent perseverates, it can only afford to occupy a limited set of states, leaving its forward model an inadequate representation of the environment.

The primary goal and purpose of the ensuing experiments is thus to illustrate, with and without boredom, (1) the extent to which an agent explores and perseverates, and (2) the forward model performance.

To this end, we motivated a model pruning hierarchy on which the comparisons above were based. The model pruning hierarchy, as summarized in Table 1 and section 5.3, provides a principled way to assess agent's behavior by progressive degrading model components. As a result, the difference between a boredom agent and a boredom-free curious agent or non-curious agent can be highlighted.

**Table 1:** Model pruning hierarchy that helps highlight the contribution of boredom and curiosity in regulating agent's exploration and perseveration.

	**Oracle**	**P/RW**	**PG/GR**	**PG/IRS**	**C/PE**	**C/B**
FM	✓	✓	✓	✓	✓	✓
AP		○	✓	✓	✓	✓
IR				○	✓	✓
VF					✓	✓
MM						✓

*Ticks mark the existence or dependence of trainable network components; circles indicate independent intervention. Top row: P/RW, random-walk policy; PG/GR, policy gradients with rewards drawn from a Gaussian distribution; PG/IRS, policy gradients with intrinsic reward samples; C/PE, curiosity using forward model error; C/B, curiosity from boredom. First column: FM, forward model; AP, action policy; IR, intrinsic rewards; VF, value function approximator; MM, meta-model*.

Explorativeness and perseveration were assessed qualitatively using Coverage Rate (CR) and Coverage Entropy (CE), reported in section 6. CR simply counts the number of states an agent has visited amongst all possible states. CE focuses on weighing the number of time steps a state was being occupied. CR thus indicates the proportion of the environment explored by the agent. Whereas, a CE curve declining over time indicates the agent tends to perseverate around a limited state space.

Forward model performance was assessed based on validation error. The validation set was sampled from the oracle dataset (see section 5.2). Contrary to self-assisted data accumulation, the oracle dataset was acquired by uniformly sampling the state-action grid. This dataset is therefore an idealized case to learn the best possible forward model.

Overall, we set the following constraints on training and environment conditions: (1) agent is responsible for assembling its own training set from scratch; (2) the probability of visiting different states is not uniformly distributed if the agent will commit to random walk; (3) the amount of time to accumulate training data points is limited.

### 5.1. Training Environment

**Figure 2:**
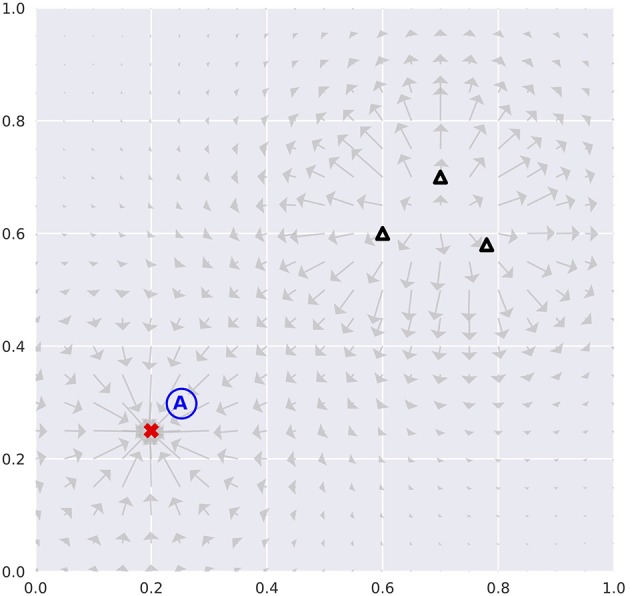
Environmental configuration. The red cross represents an attractor, whilst black triangles repellers. Vector plots indicate the forces exerted if the agent assumed the positions with zero velocities. The initial position is set at the blue letter “A.” This configuration remains identical cross all model variants and test runs.

Our agents were tested in a physics simulator, free of stochasticity, built to expand the classical Mountain Car environment (e.g., “MountainCar-v0” included in Brockman et al., 2016) into two-dimensional state space. The environment is analogous to the Mountain Car in ways that it has attractors and repellers that resemble hill- and valley-like landscapes (Figure 2). The presence of both structures serves as acceleration modifier to the agent. This makes state visitation biased toward attractors. Therefore, the acquisition of an accurate forward model necessitates planning visits to the vicinity of repellers.

The states an agent can occupy were defined as the tuple (*x, y*, ẋ, ẏ) in continuous real space. Positions (*x, y*) ∈ [0, 1]^2^ were bounded in a unit square, whereas velocities (ẋ, ẏ) were not. Boundary condition resets *x* and *y* to zero velocities. However, it is possible for the agent to slide along the boundaries if its action goes in the direction parallel to the nearby boundary. We note that being trapped in the corners is possible; though an agent could potentially get itself unstuck if appropriate actions were carried out.

Agent's action policy was represented by a categorical distribution over accelerations in *x* and *y* directions. The distribution was defined on the interval [−2.0, 2.0]^2^, evenly divided into a 11 × 11 grid. When an action is selected, the corresponding acceleration is modified according to forces exerted by the attractors and repellers.

Unlike the classical Mountain Car, our environment does not express external rewards, nor does it possess any states that are indicative of termination. Agents were allowed a pre-defined time limit (*T* = 30, 000 steps; *Data Accumulation Phase* or DAP) to act without interruption. Agent's experiences in terms of state transitions were collected in a database, which was sampled from for training at each step. During DAP, learning rates for model parameters remained constant. After DAP (or *post*-DAP), agent entered an action-free stage lasted for *T* = 30, 000, during which only sampling from own experience pool for forward model training was performed. Learning rate scheduling scheme was implemented at post-DAP.

An implementation of our training environment is available online ^1^.

### 5.2. Oracle Dataset

To contrast with self-assisted data accumulation, we constructed an oracle dataset. This dataset assumed unbiased state occupancy and action selection. We acquired the dataset by evenly dividing the state-action space into a 49 × 49 × 11 × 11 × 11 × 11 grid. Each state-action pair was passed to the physics simulator to evaluate the next state. The resultant tuple (***s***, ***a***, ***s***′) then represents one entry in the dataset. The training, testing, and validation sets were prepared by re-sampling the resulting dataset without replacement according to the ratio 0.8, 0.16, and 0.04.

A class of model referred to as Oracle, which consists of a forward model only (Table 1), was trained on this dataset. The Oracle model does not need to learn an action policy, as actions are already specified in the oracle dataset. The Oracle model was trained for 60, 000 epochs. During training, the learning rate was scheduled according to test error. Benchmarking was performed on the validation set as part of model comparisons (see section 5.4).

The oracle dataset differs from the ones that are populated by an agent as it explores. For instance, some locations in the state space are essentially inaccessible to our agent due to the force exerted by the repellers. These locations greatly inform forward model learning, however, but are only present in the oracle dataset and available to the Oracle model.

### 5.3. Model Pruning

We defined five variants of our boredom-driven curious agent. With each variation, the agent receives cumulative reductions in network components. Theses reductions are summarized as model pruning hierarchy in Table 1.

The reason that we motivated model comparisons based on model pruning is to emphasize the contribution of boredom and curiosity in regulating agent's explorativeness and perseveration. Overall, as model pruning progresses the agent was deprived of functional constructs like devaluation progress, intrinsic motivation, and planning. Eventually, the agent lost the ability to contextualize action selection and became a random-walk object. This corresponds to an ϵ-greedy policy with ϵ = 1. A random-walk agent is explorative but it cannot be considered curious in the sense that no principled means are applied to regulate explorative behaviors. With the model variants detailed below we intended to demonstrate the impact boredom and intrinsic motivation have on regulating exploration and, as a consequence, on forward model learning.

#### 5.3.1. Boredom-Driven Curiosity (C/B)

The first agent variant retained all distinctive components introduced in Section 3. The meta-model provides the devaluation progress as intrinsic rewards, whilst the value function enables the agent to plan actions that are intrinsically rewarding in the long run.

#### 5.3.2. Predictive Error-Driven Curiosity (C/PE)

The C/PE variant tests whether the induction of boredom is a constructive form of intrinsic motivation. This is achieved by removing the meta-model, thereby requiring an alternative definition of intrinsic reward. We replaced the devaluation progress with *learning progress* defined by mean squared errors of the forward model:

(17) $$\begin{array}{l} \;R_{\theta }^{\left(\ell +1\right)}:=L_{fm}(\theta^{\left(\ell \right)})-L_{fm}(\theta^{\left(\ell +1\right)})\\ L_{fm}(\theta ):=L(s^{\prime },a,s;\theta )\\ \;:=\|s^{\prime }-f(a,s;\theta ){\|}^{2} \end{array}$$

The construction of learning progress is one typical approach to intrinsic motivation and curiosity (Schmidhuber, 1991; Pathak et al., 2017).

#### 5.3.3. Policy Gradients, Intrinsic Reward Samples (PG/IRS), Gaussian Rewards (PG/GR)

Next, we examined how reward statistics alone influences policy update and, as a consequence, model learning. The value function was removed at this stage to dissociate policy learning from any downstream effects of value learning.

One distinctive feature of devaluation progress is that it entails time-varying rewards — depending on the amount of time over which an agent has evolved in the environment. We hypothesized that the emergence of curious policy is associated with reward dynamics over time. That is to say, if one perturbs the magnitudes and directions of the policy gradients with reward statistics appropriate for the ongoing time frame, the agent should exhibit similar curious behaviors. Nevertheless, we argue that such treatment is only sensible given virtually identical initial conditions. Specifically, all agent variants shared the same, environmental configuration, initial position, and network initialization.

To this end, we prepared a database for intrinsic reward samples. During C/B performance, all reward samples were collected and labeled with the corresponding time step. Afterwards, the PG/IRS agents randomly sampled from the database in a temporally synchronized manner and applied standard policy gradients.

The PG/IRS was contrasted with the PG/GR variant. Their difference lies in that a surrogate reward was used in place of the database. We defined the surrogate reward as a Gaussian distribution with time-invariant parameters, in which the mean μ = 0 is under the assumption of equilibrium devaluation progress and the standard deviation σ = 0.01, as derived from the entire database.

#### 5.3.4. Random-Walk Policy (P/RW)

Finally, we constructed a random-walk agent. All network components, apart from the forward model, were removed. This agent variant represents the case without intrinsic motivation and is agnostic to curiosity. Broadly speaking, the agent was still explorative due to its maximum entropy action policy. We regarded this version as the worse case scenario to contrast with the rest of the variants.

### 5.4. Model Comparisons

All model variants were compared on the basis of validation error given the oracle dataset. We performed 128 runs for each of the six variants (Oracle, C/B, C/PE, PG/IRS, PG/GR, and P/RW). All variants, across all runs, were assigned to identical environmental configuration (e.g., initial position, attractor/repeller placements). Network components, whenever applicable, shared identical architecture and were trained with consistent batch size and learning rate. Model parameters followed the Xavier initialization (Glorot and Bengio, 2010). During post-DAP, learning rate scheduling was implemented such that a factor 0.1 reduction was applied upon a 3000-epoch loss plateau.

## 6. Results

**Figure 3:**
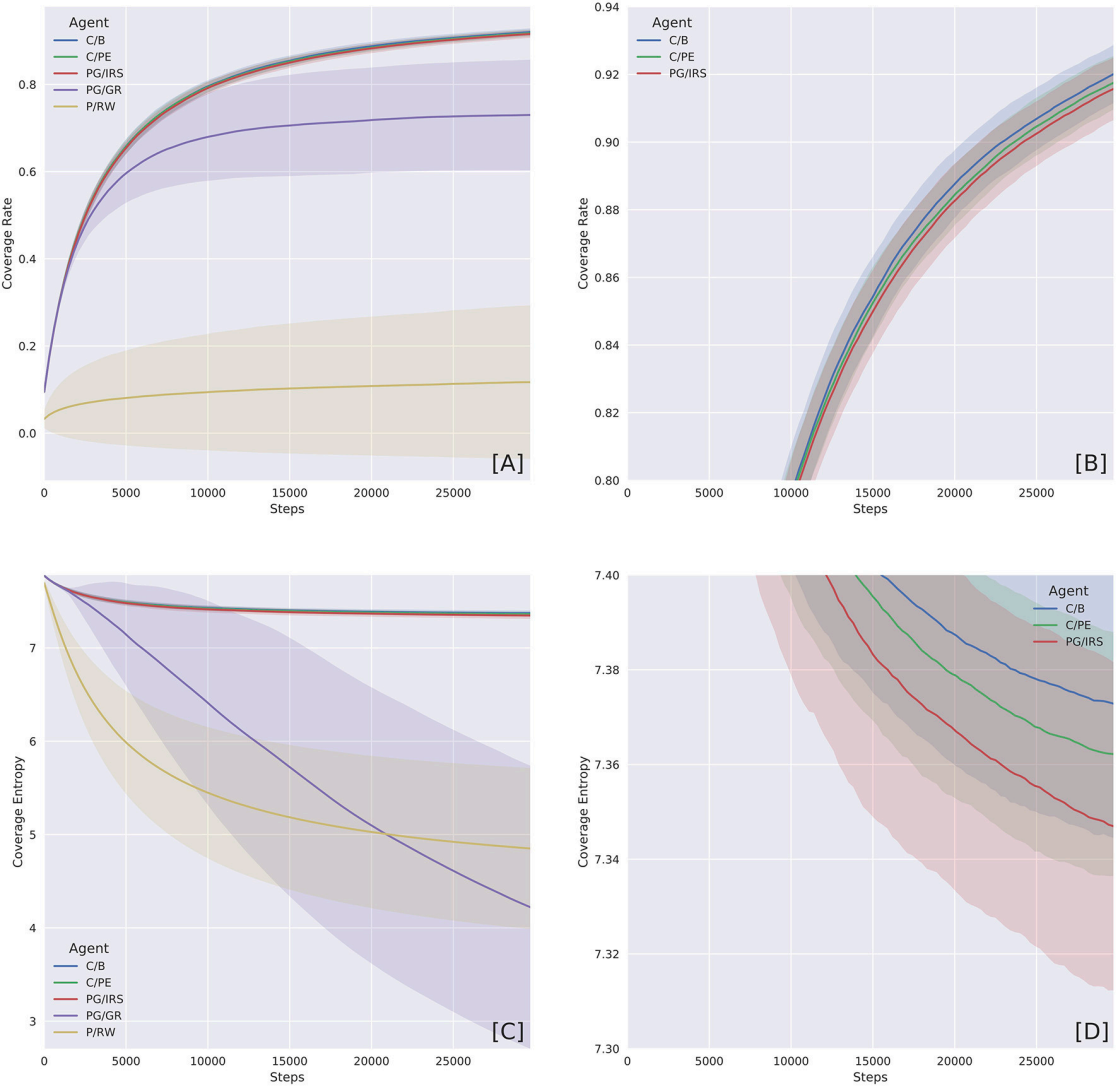
Coverage Rate (CR) and Coverage Entropy (CE) by agent variants. The two measures were computed by first turning the state space into a 50 × 50 grid, ignoring velocities. CR then marks over time whether or not a cell has been visited. Whereas, CE treats the grid as a probability distribution. Starting with maximum entropy, CR cumulatively counts the number of times a position is being visited. Entropy was calculated at each time step using the normalized counter. **(A)** Overview of CR shows the distinction between curious and non-curious agents. Curiosity caused the agents to explore faster. **(B)** Close-up on the curious agent variants, which were equally explorative. **(C)** Overview of CE shows agents with different levels of perseverance. The P/RW variants were captured by the attractor, whilst the PG/GR variants were prone to blockage. **(D)** Close-up on curious agents, which were characterized by higher CE due to attractor avoidance and more frequent repeller visitation attempts. Shaded regions represent one standard deviation.

**Figure 4:**
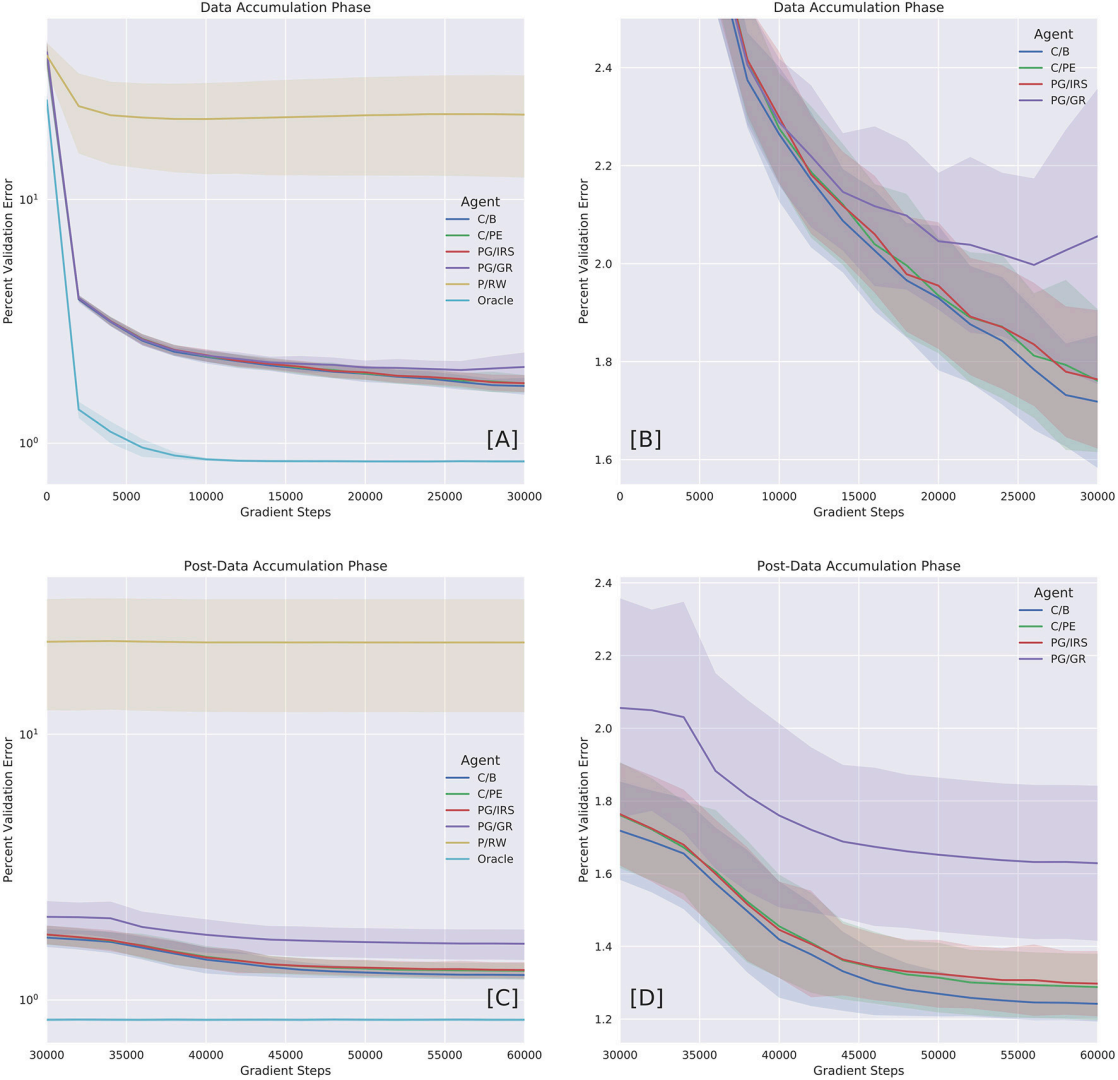
Benchmarking model variants with oracle dataset. Performances were reported in error percentage **(**also, see Table 2**)**. **(A)** Performance as a function of time during Data Accumulation Phase (DAP). **(B)** Close-up on curious variants (C/B, C/PE, and PG/IRS), as well as policy gradients (PG/GR) informed by surrogate reward statistics. The C/PE and PG/IRS variants performed similarly, but differed significantly from C/B (Table 2). **(C)** Performance over time during post-DAP. **(D)** Close-up on post-DAP performances for curious variants and PG/GR.

In this section, we offered qualitative and quantitative assessment of agent's behavioral pattern and performance across different agent variants. As established previously, an agent's performance in modeling its own environment necessarily depends on both explorative and non-perseverative behaviors. The overall picture being delivered here is that the boredom-driven curious agent (abbrev. C/B) exhibited stronger tendency toward exploration (Figures 3A,B) and against perseveration (Figures 3C,D). In accordance with our prediction, the forward model performance was significantly better for the boredom agent, as compared with other curious or non-curious variants (Figure 4 and Tables 2, 3).

**Table 2:** Summary statistics on validation loss and error percentage as benchmarking scores.

**Agent**	**DAP**		**Post-DAP**	

	**MSE loss (SD)**	**Mean Percent Error (SD)**	**MSE loss (SD)**	**Mean Percent Error (SD)**
Oracle	0.0008	0.8430	0.0008	0.8428
	(2.3E-5)	(0.0123)	(2.2E-5)	(0.0114)
C/B	0.0033	1.7181	0.0017	1.2420
	(0.0006)	(0.1357)	(0.0001)	(0.0488)
C/PE	0.0035	1.7611	0.0019	1.2882
	(0.0006)	(0.1464)	(0.0003)	(0.0916)
PG/IRS	0.0035	1.7637	0.0020	1.2976
	(0.0006)	(0.1418)	(0.0003)	(0.0902)
PG/GR	0.0048	2.0559	0.0030	1.6288
	(0.0017)	(0.3026)	(0.0008)	(0.2140)
P/RW	0.6663	22.2734	0.6615	22.1453
	(0.3904)	(10.0085)	(0.3864)	(10.0775)

*Apart from the Oracle model, a trend of declining scores can be observed as the agent degraded from C/B to P/RW, indicating the contribution of boredom and curiosity in model learning. Key: DAP, Data Accumulation Phase; SD, standard deviation. For agent codes, see Table 1*.

**Table 3:** Non-parametric statistical tests comparing terminal performance at DAP and post-DAP for curious model variants.

**Mann-Whitney ***U***-Test (*n* = 128, α = 0.025, Bonferroni corrected)**			
**Validation loss**		**DAP (*T* = 30, 000)**	**Post-DAP (*T* = 60, 000)**
C/B < C/PE	Statistics	6558.0	5911.0
	*p*-value	0.0029	5.9E-5
C/B < PG/IRS	Statistics	6275.0	5062.0
	*p*-value	0.0006	6.4E-8

*Following Table 2, even though the boredom score came close to other curious variants (C/PE and PG/IRS), the boredom variant still outperformed the other two on statistical grounds*.

We first characterized individual agent variants' qualities of being i) explorative and ii) perseverative. Active exploration is one defining attribute of curiosity (Gottlieb et al., 2013), simply because it differentiates between uncertain and known situations, thus giving rise to effective information acquisition. This, however, should be complemented with suppressed perseveration; namely, to prevent oneself from being permanently or dynamically captured—i.e., by the corners or the attractor.

The two qualities can be distinguished, as shown in Figure 3, by respective measures of Coverage Rate (CR) and Coverage Entropy (CE). The two measures were computed by first turning the state space into a 50 × 50 grid, ignoring velocities. CR keeps track of whether or not a grid cell has been visited and, at each time step, corresponds to the proportion of visited grid cells. A CR curve increasing over time indicates that an agent would be exploring new grid cells.

CE, on the other hand, accounts for the number of time steps an agent revisited one grid cell. This then gives an empirical probability distribution at each time step that reports the likelihood of finding an agent occupying a grid cell. A concentrated probability distribution means an agent only paid visit to a small set of grid cells and, as a result, the probability distribution has low entropy.

Because (state) visitation bias was inherent in our training environment, naturally, agents occupying a subset of states would cause CE to reduce faster than those who attempted to escape. The C/B, C/PE, and PG/IRS variants were regarded as curious and intrinsically motivated. Our results showed that these variants were predominantly explorative and non-perseverative. By contrast, the P/RW agent, albeit explorative, had no principled means to escape the potential well. However, if *t* → ∞ the P/RW should be able to explore further by chance. The PG/GR variant, on the other hand, exhibited, intermediate explorativeness and extreme perseverance with disproportionately high variance. We attributed this behavior to the detrimental effects of inappropriately informative reward statistics.

Next, we benchmarked forward model performance of individual variants by their validation loss and error percentage. We reported DAP and post-DAP performances separately as a function of time in Figure 4. Error percentage was calculated as the percent ratio between root mean squared loss and the maximum pair-wise Euclidean distance in the validation set. This ratio can be summarized by $∥{\text{s}}_{k}^{\prime }-f\left({\text{a}}_{k},{\text{s}}_{k};\theta \right)∥/\underset{i,j}{\max}∥D_{i}-D_{j}∥$, where D is the validation set and $\left({\text{s}}_{k}^{\prime },{\text{a}}_{k},{\text{s}}_{k}\right)\in D$.

The Oracle model, trained under the supervision of oracle training set, reached an error percentage of 0.84% for both DAP and post-DAP, amounting to approximately 30% improvement over the terminal performance of the C/B variant. All variants considered curious (C/B, C/PE, and PG/IRS) had similar performances during DAP. In particular, the PG/IRS, which received independent intervention from the ‘trueș reward distributions achieved marginally lower performance but indistinguishable from the C/PE variant. This outcome was observed for both DAP and post-DAP, suggesting intrinsic reward samples derived from C/B contributed favorably even to the standard policy gradients algorithm.

Though without the ability to approximate value function, the PG/IRS variant underperformed in benchmarking, as compared with the value-enabled, C/B variant. Using non-parametric test, the difference was detected for DAP (*p* = 0.0006) and post-DAP (*p* = 6.4E-8), respectively. Similar observations were also made for comparisons between C/B and C/PE, at *p* = 0.0029 (DAP) and *p* = 5.9E-5 (post-DAP). Overall, this suggested significant differences in the experiences accumulated across agent variants. The aforementioned statistics were reported in Tables 2, 3.

## 7. Limitation

One obvious limitation of the proposed method is scalability. We imposed Gaussian assumption on the forward model and meta-model because this lends the KL-divergence between the two to have a closed form solution. However, this solution depends on both matrix inversion and log-determinant, whose computational complexity normally falls around an order of 3 when using Cholesky decomposition. To circumvent this limitation, the intrinsic reward (devaluation progress) may be replaced with one based on (forward model) prediction error at the expense of lesser curiosity.

The Gaussian assumption also puts limitations on the expressiveness of the models. This can be slightly relaxed to admit Gaussian mixture models. KL-divergence between Gaussian mixture models is not tractable but can nonetheless be approximated (e.g., Hershey and Olsen, 2007). Alternatively, employing normalizing flows (Rezende and Mohamed, 2015) also allows expressive models. Calculating KL-divergence in this case is typically resorted to Monte Carlo approximation. These are potential extensions that can be applied to the current work in the future.

## 8. Conclusion

We have provided a formal account on the emergence of boredom from an information-seeking perspective and addressed its constructive role in enabling curious behaviors. Boredom thus motivates an instrumental view of action selection, in which an action serves to disclose outcomes that have intrinsic meaningfulness to an agent itself. This is, a bored agent must seek out information worth assimilating into itself. This led to the central claim of this study—pertaining to the superior data-gathering efficiency and hence effective curiosity. We supported this claim with empirical evidence, showing that boredom-enabled agents consistently outperformed other curious agents in self-assisted forward model learning. Our results solicited the interpretation that the relationship between homeostatic and heterostatic intrinsic motivations can in fact be complementary; therefore, we have offered one unifying perspective for the intrinsic motivation landscape.

Our proposed method is general in formalization and sits comfortably with existing MDP problems. Our future work is then to apply the method to more complex problems, such as embedding into a robot for real-world scenarios.

## Author Contributions

YY conceived of this study, performed the experiments, and wrote the first draft of the manuscript. AC programmed the physics simulator, wrote part of Introduction, and created Figure 1. All authors contributed to manuscript revision, read and approved the submitted version.

### Conflict of Interest Statement

The authors declare that the research was conducted in the absence of any commercial or financial relationships that could be construed as a potential conflict of interest.

## Footnotes

^1^https://github.com/arayabrain/MountainCar2D
